# Competitive release of drug resistance following drug treatment of mixed *Plasmodium chabaudi *infections

**DOI:** 10.1186/1475-2875-3-33

**Published:** 2004-09-14

**Authors:** Jacobus C de Roode, Richard Culleton, Andrew S Bell, Andrew F Read

**Affiliations:** 1 Institutes of Evolution, Immunology and Infection Research, School of Biological Sciences, University of Edinburgh, King's Buildings, West Mains Road, Edinburgh EH9 3 JT, Scotland, United Kingdom

## Abstract

**Background:**

Malaria infections are often genetically diverse, potentially leading to competition between co-infecting strains. Such competition is of key importance in the spread of drug resistance.

**Methods:**

The effects of drug treatment on within-host competition were studied using the rodent malaria *Plasmodium chabaudi*. Mice were infected simultaneously with a drug-resistant and a drug-sensitive clone and were then either drug-treated or left untreated. Transmission was assessed by feeding mice to *Anopheles stephensi *mosquitoes.

**Results:**

In the absence of drugs, the sensitive clone competitively suppressed the resistant clone; this resulted in lower asexual parasite densities and also reduced transmission to the mosquito vector. Drug treatment, however, allowed the resistant clone to fill the ecological space emptied by the removal of the sensitive clone, allowing it to transmit as well as it would have done in the absence of competition.

**Conclusion:**

These results show that under drug pressure, resistant strains can have two advantages: (1) they survive better than sensitive strains and (2) they can exploit the opportunities presented by the removal of their competitors. When mixed infections are common, such effects could increase the spread of drug resistance.

## Background

Malaria infections often consist of more than one parasite genotype [1-3]. Humans represent ecological niches for co-infecting malaria parasites, with shared predators (immune responses) and limited resources, so that competition between co-infecting malaria strains is likely to be intense [4]. Such competition could strongly affect the relative transmission of newly arisen drug-resistant strains, and thus the spread of drug resistance [5].

Resistant and sensitive strains will co-occur in the same host both when *de novo *mutations arise, and when hosts acquire resistant and sensitive strains from one mosquito bite simultaneously or from different mosquito bites. In the absence of drug treatment, the transmission success of the resistant strain will depend on its intrinsic fitness and competitive ability. However, if drug treatment does occur, the resistant strain has two potential fitness advantages. First, it will better survive the drug than the sensitive strain. Second, treatment can remove drug-sensitive competitors, thus freeing up ecological space for the resistant strains to occupy; this would increase the relative transmission of the drug-resistant strain. This second effect, well recognized in theory, has the potential to greatly enhance the rate of spread of drug resistance in a population [5]. However, there is no direct experimental evidence that removal of competitors by drug treatment does enhance the transmission of drug-resistant parasites. This paper reports the first direct experimental demonstration that competitive release of drug-resistant strains can occur following drug treatment.

Competition between drug-sensitive and -resistant malaria clones was studied using the rodent malaria *Plasmodium chabaudi*. This parasite is commonly used as a model for human malaria [6], and has been extensively used to study drug resistance [7]. In the absence of drugs, the drug-resistant clone is competitively suppressed by a drug-sensitive clone [8]. Here, competition between the two strains in drug-treated and untreated mice is compared.

## Methods

Two genetically distinct *Plasmodium chabaudi chabaudi *clones were used: an AS clone resistant to the antifolate drug pyrimethamine [9], and AJ, a sensitive clone. These clones will be referred to as R (for resistant) and S (for sensitive) from hereon. Hosts were eight weeks old CBA/Ca inbred female mice (Ann Walker, University of Edinburgh; Harlan, England). Two experiments were performed. In the first, two groups of five mice were infected with 10^6 ^R parasites, and two groups with 10^6 ^R + 10^6 ^S parasites, as described elsewhere [8]. One group from each of these two infection types was drug-treated within three hours of inoculation and again on days 1, 2 and 3 PI (post-infection), using an oral administration of 8 mg pyrimethamine per kg mouse body weight.

Asexual parasite densities and gametocyte densities – the latter being the transmission stages to the mosquito – were monitored using microscopic examination of thin blood smears and determination of red blood cell densities using flow cytometry (Beckman Coulter), as described elsewhere [8]. Real-time quantitative PCR was used to distinguish and quantify R and S parasites in mixed infections [8,10]. This protocol cannot distinguish between asexual parasites and gametocytes, but real-time PCR data were used as estimates of asexual densities, because gametocyte densities were 2–3 orders of magnitude lower than asexual densities and thus a negligible component of overall parasite numbers. For each infection, two phases were distinguished: the acute phase, involving the first wave of parasites, and the chronic phase, beginning when parasite numbers began to recover after the collapse of that first wave around day 12. All parasites had disappeared below detectable levels after 50 days.

In the second experiment, two groups of nine mice were infected as above with either R parasites or R+S parasites. The subsequent transmission success of clone R was assayed by allowing batches of 30 starved *Anopheles stephensi *mosquitoes to feed on 3 mice from each group on each of days 7, 14, and 21 PI, as described elsewhere [e.g. [11]]. Eight days after the feeds, mosquitoes were dissected, and DNA extracted from midguts carrying oocysts. Real-time quantitative PCR was subsequently used to determine the prevalence of clone R in these mosquitoes.

All procedures were regulated and carried out under the British Home Office Animals (Scientific Procedures) Act 1986.

## Results

Two untreated mice infected with R+S parasites died on days 10 and 11 PI respectively, and were excluded from the analysis.

**Figure 1:**
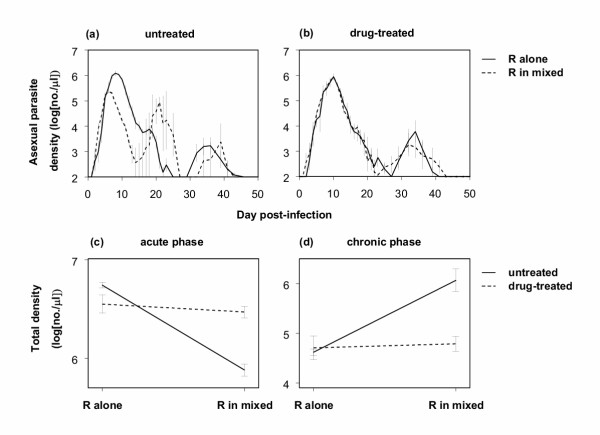
Log asexual parasite densities of the resistant clone R over time in untreated (a) and drug-treated (b) mice infected with R alone or a mixture of R+S clones, and total numbers of R parasites produced over the acute (c) and chronic phases (d). All data points (mean ± 1 s.e.m.) are based on 5 replicate mice, except for mixed infections in untreated mice in (a) (4 mice on day 11 and 3 mice from day 12 onwards) and (c) and (d) (3 mice). As the limit of detection was 100 parasites per μl blood, y-axes in (a) and (b) start at 2.

In untreated mice, there were far fewer R parasites during the acute phase in mixed infections with clone S than in R-only infections (Figures 1a,1c). However, in drug-treated mice, where S parasites were entirely removed by pyrimethamine (none of the PCR reactions performed were positive for clone S), there were as many R parasites in mixed infections as there were in R-only infections (Figures 1b,1c; Drug treatment × Alone/Mixed interaction: F_1,14 _= 14.4, p = 0.002). Thus, R parasites were competitively suppressed in mixed infections in untreated mice, but this suppression was negated when mice were treated with pyrimethamine, which effectively removed S parasites.

During the chronic phase, clone R was more numerous in untreated mice in mixed infections than in single-clone infections (due to the parasite peak around day 21; Figures 1a,1d). Thus, in untreated mice in the chronic phase, clone R did not suffer from competition, and actually benefited from the presence of clone S (facilitation). In drug-treated mice, however, R parasites were similarly numerous in mixed- and single- clone infections (Figures 1b,1d; Drug treatment × Alone/Mixed interaction: F_1,14 _= 13.8, p = 0.002).

**Figure 2:**
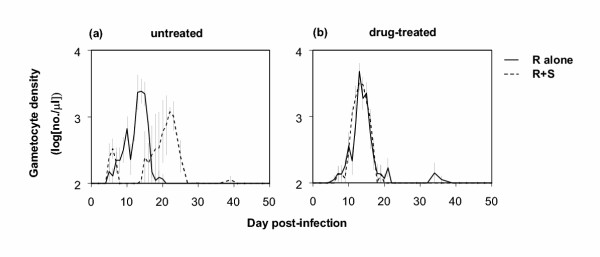
Log gametocyte densities over time (mean ± 1 s.e.m.) for untreated (a) and drug-treated (b) mice. In (a) gametocyte densities for mixed R+S infections reflect overall R+S gametocytes, as the PCR assay could not distinguish between these (see text); in (b) all gametocytes are produced by clone R, as clone S was cleared from mixed infections. All data points are based on 5 replicate mice, except for mixed infections in untreated mice in (a): 4 mice on day 11 and 3 mice from day 12 onwards. As the limit of detection was 100 gametocytes per μl blood, y-axes start at 2.

The large peak of R parasites in the chronic phase in the untreated mixed infections around day 21 (Figure 1a) coincided with a large peak of gametocytes, the transmissible stages of the parasite (Figure 2a). This was in contrast with single-clone infections of R in untreated mice, and infections in drug-treated mice, where gametocytes were mainly produced around day 14 (Figures 2a,2b). Overall, gametocyte numbers were the same for all four infection types (p > 0.05 for both Drug treatment and Alone/Mixed). Whether clone R really suffered from competitive suppression by clone S in untreated mice thus depends on how many of the gametocytes around day 21 were of the R genotype, and on how transmissible they were.

**Figure 3:**
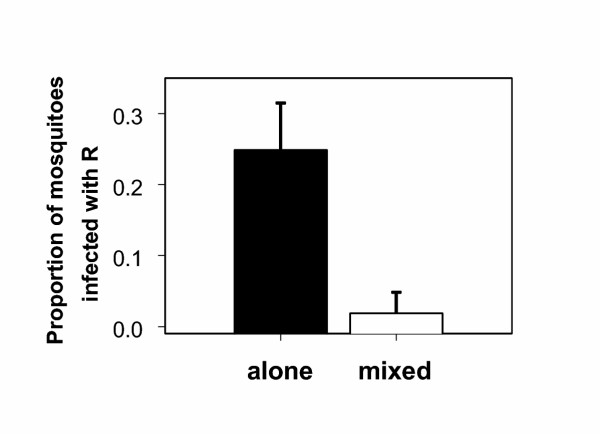
Proportions of mosquitoes infected with the resistant clone R (mean and 95% confidence interval); mosquitoes fed either on mice infected with clone R alone or mice infected with a mixture of clones R and S. Means are based on 9 mice (3 on day 7, 3 on day 14 and 3 on day 21 PI) from which totals of 205 (R alone) and 216 (mixed R+S) mosquitoes took a blood meal. Infection with clone R was assessed by real-time PCR.

The second experiment assessed transmission to mosquitoes on days 7, 14 and 21 PI. It was found that the resistant clone R infected far fewer mosquitoes from mixed infections than from single infections (figure 3; Alone/Mixed: p = 0.002), indicating that transmissibility of gametocytes produced around day 21 was low, probably as a result of transmission-blocking immunity [12]. Thus, the competitive suppression of the resistant clone in untreated infections translated into reduced transmission success.

## Discussion

These results show that drug treatment of malaria infections can severely affect ecological interactions between co-infecting strains. The drug-resistant clone was competitively suppressed by the drug-sensitive clone in untreated mice, in terms of both within-host growth and transmission to the mosquito vector. However, drug treatment removed that competitive suppression, and allowed the resistant clone to fill the ecological space emptied, giving it a substantial and additional fitness benefit in addition to the simple survival advantage conferred by resistance. Thus, under drug pressure, resistant strains can have two advantages: they survive better than sensitive strains and they can exploit the opportunities presented by the removal of their competitors, thereby increasing their relative transmission. Competition was studied between two unrelated clones, and thus did not reflect the situation in which a resistant clone arose *de novo *[13], but it seems likely that the competitive release following drug therapy would also apply there.

Competitive release following drug treatment will greatly enhance the spread of drug resistance [5]. Also, with only the resistant strain left in the host, the probability of outbreeding is reduced, thus reducing the chances of meiotic recombination destroying multi-locus resistance [14]. In combination, these two processes could enhance the spread of drug resistance, especially in areas with high numbers of strains per infection [5].

Of course, this is an argument for judicious use of drugs, not their non-use. Clearance of drug-sensitive strains from mixed infections might enhance the spread of drug resistance, but this has to be offset against the short-term public health benefits, such as reducing overall malaria prevalence. In these experiments, the drug-sensitive clone was also the more virulent clone [8], and when it was cleared from mixed infections by drug treatment, mice were less sick, in that they lost less weight and became less anaemic (results not shown).

In this experiment, mice were drug-treated before symptoms occurred, resulting in competitive release. This situation perhaps best mimics the case of prophylactic drug use, or what might occur to new co-infections in high transmission areas where drug use is common. A battery of more complex experiments will be necessary to determine if competitive release occurs when treatment follows symptoms, and when drugs are used to treat semi-immune individuals. The facilitation observed in chronic infections (Figures 1a,1d) suggests the situation might be very complex.

Within-host competition in *P. chabaudi *is now firmly established [8,15,16]. Evidence for competition between co-infecting genotypes in human malaria infections is necessarily indirect, but consistent with this [4]. In older children and adults, for example, parasite densities do not increase with increasing numbers of clones, thus indicating that parasite clones are not regulated independently [17]. Given this, and the high frequency of mixed infections in human malaria [1-3,18] often consisting of both resistant and sensitive genotypes [19], and the fact that genetic diversity can be altered by antimalaria prophylaxis [20], it is highly likely that competitive release of drug resistance also occurs in human malaria. Indeed, a recent study has already implicated release of within-host competition as a key-factor in the spread of drug resistance in Uganda [21].

## Authors' contributions

JCdR and RC designed and performed the first experiment, while JCdR and ASB performed the second experiment. JCdR analysed the results and drafted the manuscript. ASB developed the real-time PCR assays for analysis of parasite populations inside mosquitoes. AFR assisted in designing both experiments and writing the manuscript. All authors read and approved of the final version of the manuscript.

## Competing interests

None declared.
